# Repeated Herbal Acupoint Sticking Relieved the Recurrence of Allergic Asthma by Regulating the Th1/Th2 Cell Balance in the Peripheral Blood

**DOI:** 10.1155/2020/1879640

**Published:** 2020-05-17

**Authors:** Shu-Mei Zhao, He-Sheng Wang, Cong Zhang, Jun Hu, Lin-Li Zhuang, Xing Wang, Yu-Qian Fan, Wen-Jiao Hu, Jia-Qi Luo, Ning-Wei Zhao, Shi-Hai Yan, Jie Dong, Lan-Ying Liu, Qian Lu, Meng Cao

**Affiliations:** ^1^Affiliated Hospital of Nanjing University of Chinese Medicine, Nanjing 210029, China; ^2^Nanjing University of Chinese Medicine, Nanjing 210029, China; ^3^Medical School of Nanjing University, Nanjing 210093, China; ^4^The Affiliated Brain Hospital of Nanjing Medical University, Nanjing 210029, China; ^5^Affiliated Hospital of Integrated Traditional Chinese and Western Medicine Nanjing University of Chinese Medicine, Nanjing 210028, China

## Abstract

Allergic asthma is an inflammatory disease involving the Th1/Th2 cell imbalance in the peripheral blood. Repeated herbal acupoint sticking (RHAS) has been used for hundreds of years in China to relieve the recurrence of allergic asthma, and it is still practiced today. Thus, we explored the effect on allergic asthma relapse and the underlying immunoregulatory mechanism in this study. Here, we enrolled 50 allergic asthma participants, and 38 of them completed the treatment and follow-up (the allergic asthma group). In addition, 13 healthy participants (the control group) were enrolled. The recurrence number of allergic asthma participants and asthma control test (ACT) were used to evaluate the effect of treatment on relieving allergic asthma recurrence. Flow cytometry was performed to analyze the levels of Th1 and Th2 cells in the peripheral blood. The serum levels of IgE, IFN-*γ*, and IL-4 were detected by ELISA. (1) In the allergic asthma group, compared to before the first treatment, the recurrence number of allergic asthma participants decreased and the ACT score increased at end of the last treatment, 18 and 30 weeks of the trial (*P* < 0.05). At 18 and 30 weeks of the trial, the recurrence number of allergic asthma participants was less and the ACT score was higher than the ones from the same period last year in the allergic asthma group (*P* < 0.05). Compared to before the first treatment, the percentage of Th1 cell did not change significantly, the percentage of Th2 cell decreased, and the Th1/Th2 cell ratio increased in the allergic asthma group by the end of the last treatment (*P* < 0.05). Meanwhile, the release of IgE and IL-4 reduced (*P* < 0.05), and the release of IFN-*γ* did not significantly change in the allergic asthma group. (2) Compared with the control group, the serum levels of IgE and IL-4 and the percentage of Th2 cell were higher, and the Th1/Th2 cell ratio was lower in the allergic asthma group (*P* < 0.05). There was no significant difference between Th1 cell and IFN-*γ* before the first treatment. (3) Compared with the control group, the IgE levels and the percentage of Th2 cell were higher in the allergic asthma group (*P* < 0.01). Simultaneously, there was no significant difference between Th1 cell, the Th1/Th2 cell ratio, and the serum levels of IFN-*γ* and IL-4 by the end of the last treatment. The data suggested that RHAS reduced the amount of Th2 cell and elevated the Th1/Th2 cell ratio, thereby alleviating the inflammatory responses in the allergic asthma participants.

## 1. Introduction

Allergic asthma is an IgE-mediated airway disease caused by repeated exposure to allergens [1]. It is characterized by chronic airway inflammation caused by a variety of immune cells, especially T cells [2], which is closely related to the imbalance of Th1/Th2 cell and their cytokines [3]. Th2 cell initiates the immune response of allergic asthma by releasing type 2 cytokines, such as IL-4 that promotes the synthesis of IgE by B cells [4]. Compared to Th2 cell, type 1 cytokines, say, IFN-*γ*, is involved in antagonizing Th2 cell response and IgE synthesis to inhibit allergic asthma progression [5]. Therefore, an effective therapy for allergic asthma is to stimulate Th1 cell while simultaneously suppressing the Th2 cell immune response to regulate the balance of Th1/Th2 cell [6]. At present, drug therapies for allergic asthma are mainly inhaled glucocorticoids, which can alleviate the clinical symptoms of many patients, but excessive dependence on glucocorticoids can produce pharyngeal ulcers, osteoporosis, and other side effects [7, 8]. These issues highlight the need for more clinically effective allergic asthma interventions.

RHAS is an effective traditional Chinese medicine treatment, carried out in the summer to relieve the recurrence of allergic asthma that has been used in China for hundreds of years. Wen et al. [9] evaluated 18 studies involving 1785 subjects conducted before 2015 and indicated that RHAS had been widely and successfully used for the treatment of allergic asthma. Wu et al. [10] studied the RHAS treatment of allergic asthma patients for two consecutive years; the frequency and severity of allergic asthma recurrence reduced, and the number of medications used also decreased significantly.

Therefore, our present study explored the immunoregulatory mechanism of RHAS by studying the levels of IgE, Th1 and Th2 cells, IFN-*γ*, and IL-4. Our findings will enable a more efficacious treatment of allergic asthma using a convenient modality than conventional drug regimes, which has few side effects. This will also improve our understanding of the specific mechanism of allergic asthma and its response to traditional Chinese medicine.

## 2. Materials

### 2.1. Trial Information

**Figure 1:**
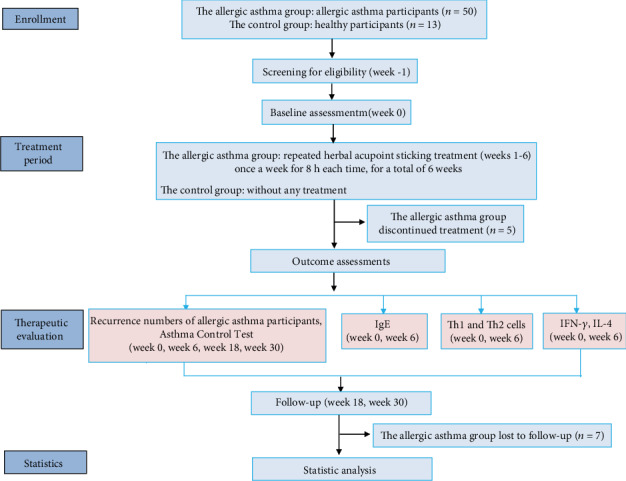
The schematic design of the present trial.

The details of the clinical controlled trial are presented in Figure 1. The trial consisted of three phases: a 1-week screening period (week 1), a 6-week RHAS treatment period (weeks 1 to 6), and a 24-week follow-up period (weeks 7 to 30). The recurrence number of allergic asthma participants was obtained, and ACT was performed at 0, 6, 18, and 30 weeks [11]. At 0 and 6 weeks, the venous blood was collected to detect the levels of IgE, Th1 and Th2 cells, IFN-*γ*, and IL-4.

### 2.2. Participants and Safety Information

The study was approved by the Affiliated Hospital of Nanjing University of Chinese Medicine Ethics Committee (2017NL-11-02). All participants provided written informed consent (trial registration ChiCTR1800016644).

Allergic asthma participants were recruited from the Affiliated Hospital of Nanjing University of Chinese Medicine from June to August 2018. Asthma diagnosis was based on the 2014 revision GINA guideline [12], who, with a confirmed IgE sensitization to at least one airborne perennial allergen in clinical remission aged 18 to 75 years, were included. In addition, eligible participants all had a physician's diagnosis and experienced chronic persistent symptoms. Through in-person interviews, researchers obtained information about age, sex, and the course of disease. A total of 50 allergic asthma participants were enrolled in the allergic asthma group, and twelve of them lost (five dropped out during treatment and seven failed to complete the follow-up). Therefore, the 38 remaining eligible allergic asthma participants (14 male and 24 females, average age of 52.2 years) were included in the study. In addition, we recruited 13 healthy participants (4 male and 9 females, average age of 50.9 years) from Nanjing, Jiangsu Province, as the control group, as shown in Table 1.

During the clinical trial, we undertook a series of measures to monitor possible adverse events and side effects. Schedule of study procedures is shown in Table 2. Special focus was placed on monitoring potential liver and kidney damage to assess safety from the time of enrolment to the end of the follow-up period. Laboratory tests were conducted on the blood, as well as kidney and liver function, at the time of enrolment and end of the last treatment. During each visit, participants were asked about any adverse effects, regardless of the intervention method used. In case of serious adverse events, treatment was immediately stopped, and allergic asthma participants were appropriately treated based upon the symptoms of their adverse reaction.

### 2.3. Composition and Preparation Process for RHAS Material

*Semen Brassicae*, *Corydalis tuber*, *Euphorbia kansui*, *Asarum*, *Syzygium aromaticum*, *Fructus Gleditsiae*, *Lepidium* seed, *Cinnamomum*, and *Ephedra* (provided by the pharmacy department of Affiliated Hospital of Nanjing University of Chinese Medicine) were powdered and sifted with size 80 mesh sieves then mixed thoroughly in a clean container at a ratio of 2 : 2 : 1 : 1 : 1 : 1 : 1 : 1 : 1, respectively. 70 ml fresh ginger juice (provided by the pharmacy department of the hospital) and 30 ml of Vaseline were heated to a liquid state at 60°C added to 110 g of mixed powder. The result was used to prepare a circular patch with a 2 cm diameter and 1 mm thickness, weighing approximately 1.5 g.

## 3. Methods

### 3.1. Treatment

In the allergic asthma group, researchers fully exposed the participant's back, dried any local sweat, and then bilaterally attached the herbal acupoint sticking patch directly to the skin of *Dingchuan* (extrameridian points B1), *Feishu* (the bladder meridian of foot-taiyang 13), *Xinshu* (the bladder meridian of foot-taiyang 15), *Pishu* (the bladder meridian of foot-taiyang 20), and *Shenshu* (the bladder meridian of foot-taiyang 23) points. Finally, the researchers used 5 cm × 5 cm desensitization tape to externally fix the patches. Patches were attached once a week for 8 h each time, for a total of 6 weeks. In the control group, healthy participants did not receive any treatment.

### 3.2. Main Determination Methodology

#### 3.2.1. Recurrence Number of Allergic Asthma Participants

It mainly depended on whether the allergic asthma participants had a relapse. Before the first treatment, researchers enquired the participants and asked to recall the recurrence number of allergic asthma participants about the same period last year of 18 and 30 weeks of the trial by face to face, and then they recorded it. It was enquired and recorded again at the end of the last treatment. At 18 and 30 weeks, the participants were interviewed by phone.

#### 3.2.2. ACT Score

The method of obtaining ACT score was the same as that of recurrence number of allergic asthma participants. ACT was used for scoring and year on year comparison. ACT contained five questions, and each question was assessed by five scores (a total of 25 scores). A score of 25 showed full control, 20 to 24 scores showed partial control, and <20 scores showed uncontrolled asthma. Higher score denoted better allergic asthma-related quality of life.

#### 3.2.3. Flow Cytometric Analyses of Th1 and Th2 Cells

The venous blood (2 ml) was taken and placed in an anticoagulant tube before the first treatment and at the end of the last treatment. Researchers placed 200 *μ*l of the anticoagulant venous blood and 200 *μ*l of 1640 medium in each tube and then added 0.8 *μ*l cell stimulation cocktail plus protein transport inhibitors (BioLegend, San Diego, CA, USA). The tube was incubated at 37°C for 4-6 h. After removing the tube, 1 ml of RBC lysis buffer was added. This step was repeated and then washed once with cell staining buffer (BioLegend, San Diego, CA, USA). Then, 4 *μ*l CD3 antibody (FITC, BioLegend, San Diego, CA, USA) and 4 *μ*l CD8a antibody (PerCP/Cy5.5, BioLegend, San Diego, CA, USA) were added at 4°C for 30 min in the dark to mark cells. The fixation buffer (500 *μ*l; BioLegend, San Diego, CA, USA) was added to the tube and then the cells were fixed at room temperature for 30 min in the dark. The cell membranes were ruptured twice using an intracellular staining perm wash buffer (BioLegend, San Diego, CA, USA). Then, 4 *μ*l IL-4 antibody (PE, BioLegend, San Diego, CA, USA) and 4 *μ*l IFN-*γ* antibody (APC, BioLegend, San Diego, CA, USA) were added to the tube to mark the cells. Intracellular staining was performed at 4°C for 30 min in the dark. Tube contents were washed with cell staining buffer and analyzed by flow cytometry (BD Biosciences, San Jose, CA, USA).

#### 3.2.4. ELISA Detection of IgE, IFN-*γ*, and IL-4

The serum levels of IgE, IFN-*γ*, and IL-4 were measured using ELISA according to the manufacturer's instructions (KeyGen Biotech, Nanjing, China). The venous blood (3 ml) was taken and placed in a procoagulant tube before the first treatment and again at the end of the last treatment. After centrifugation at 1000 g for 15 min, 1 ml of serum was collected and stored at -80°C. The serum samples (100 ml per well) were added into 96-well plates. The biotinylated antibodies were added to each well and incubated at 36°C for 90 min. The wells were aspirated and then each well was washed five times. The substrate solutions were added to each well and then incubated for 30 min at 36°C in the dark. The optical density of each well was determined using a microplate reader (BioRad Model 680, USA) that was set to 450 nm, and the reading results were saved in the instrument.

### 3.3. Statistical Analysis

Before the first treatment, measurements of age, sex, course of disease, recurrence number of allergic asthma participants, ACT score, IgE, Th1 and Th2 cells, IFN-*γ*, and IL-4 were used to calculate the baseline levels for each participant. Continuous variables were expressed as mean ± standard error of mean (M ± SEM) with a paired-samples *t*-test and two independent samples *t*-test for parametric data and Wilcoxon's Signed-Rank tests and Mann–Whitney *U* tests for nonparametric data. *P* < 0.05 indicates statistical difference, and *P* < 0.01 indicates that each data item has significant difference. The IBM SPSS Statistics for Windows, Version 25.0 (IBM Corp.), software was used for analyzing the clinical parameters. The plot was drawn with the GraphPad Prism 5 software (GraphPad Software).

## 4. Results

### 4.1. RHAS Decreased the Recurrence Number of Allergic Asthma Participants

(1) Compared to before the first treatment, the recurrence number of the allergic asthma group decreased both at end of the last treatment (*P* = 0.016) and 18 (*P* = 0.031) and 30 (*P* = 0.031) weeks of the trial. (2) Compared to the same period of the previous year, the recurrence number of the allergic asthma group decreased both at 18 (*P* = 0.031) and 30 (*P* = 0.001) weeks of the trial, as shown in Table 3.

### 4.2. RHAS Increased the ACT Score

**Figure 2:**
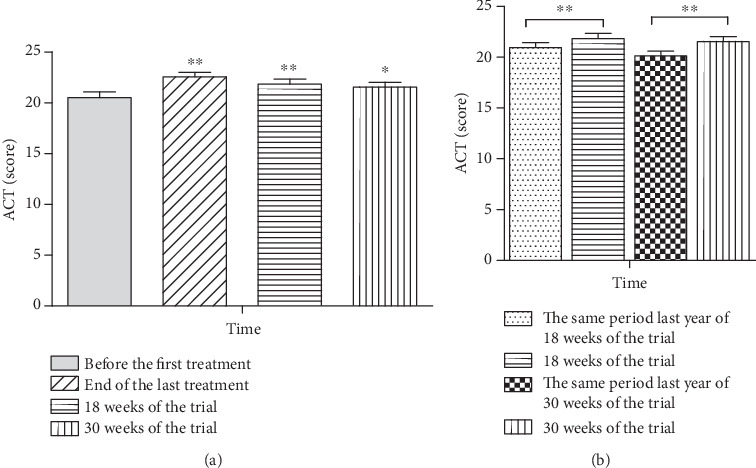
Effect of repeated herbal acupoint sticking on the ACT score of allergic asthma participants. (a) Compared to before the first treatment, the changes of the ACT score at the end of the last treatment and 18 and 30 weeks of the trial. (b) Compared to the same period last year of 18 and 30 weeks of the trial, the changes of ACT score at 18 and 30 weeks of the trial. Data are expressed as M ± SEMs, analyzed by the nonparametric Wilcoxon Signed-Rank Test. ^∗∗^*P* < 0.01, ^∗^*P* < 0.05.

(1) Compared to the baseline score, the ACT score of the allergic asthma group increased both at end of the last treatment (*P* = 0.001) and 18 (*P* = 0.002) and 30 (*P* = 0.02) weeks of the trial. (2) Compared to the same period of the previous year, the ACT score of the allergic asthma group increased both at 18 (*P* = 0.003) and 30 (*P* = 0.001) weeks of the trial, as shown in Figure 2.

### 4.3. RHAS Inhibited the Release of IgE

**Figure 3:**
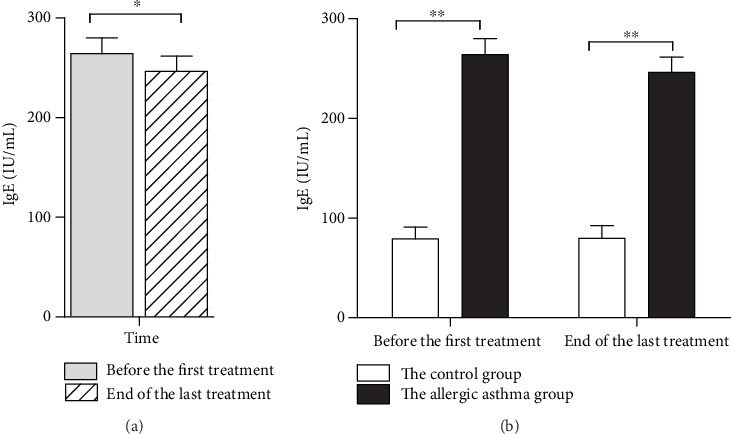
Effect of repeated herbal acupoint sticking of IgE of participants. (a) The release of IgE before the first treatment and at the end of the last treatment in the allergic asthma group. (b) The release of IgE of the control group and the allergic asthma group before the first treatment and at the end of the last treatment. Data are expressed as M ± SEMs (the control group: *n* = 13, the allergic asthma group: *n* = 38), analyzed by the paired-samples *t*-test intragroup comparison and nonparametric Mann–Whitney *U* test intergroup comparison. ^∗∗^*P* < 0.01, ^∗^*P* < 0.05.

(1) It was shown that RHAS treatment reduced the serum levels of IgE at end of the last treatment (*P* = 0.02). (2) Compared with the control group, the IgE levels of the allergic asthma group were higher before the first treatment and at the end of the last treatment (*P* = 0.001), as shown in Figure 3.

### 4.4. RHAS Regulated the Ratio of Th1 and Th2 Cells

**Figure 4:**
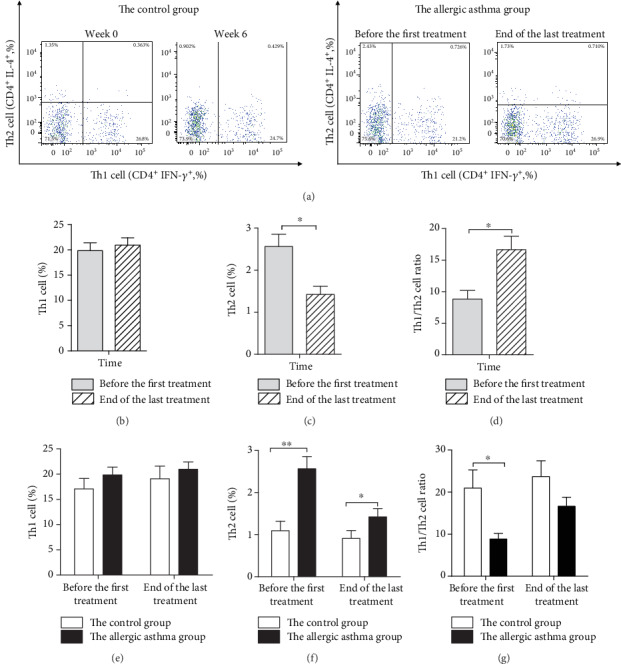
Effect of the repeated herbal acupoint sticking on Th1 andTh2 cells among CD4^+^ T cells in the peripheral blood of participants. (a) Flow cytometric analysis of the peripheral blood stained successively with anti-CD3 (FITC), anti-CD8a (PerCP/Cy5.5), anti-IFN-*γ* (PE), and anti-IFN-*γ* (APC). Because the control group had no treatment, the time point of detection was at weeks 0 and 6. (b) The percentage of Th1 cell before the first treatment and at the end of the last treatment in the allergic asthma group. (c) The percentage of Th2 cell before the first treatment and at the end of the last treatment in the allergic asthma group. (d) The Th1/Th2 cell ratio before the first treatment and at the end of the last treatment in the allergic asthma group. (e) The percentage of Th1 cell of the control group and the allergic asthma group before the first treatment and at the end of the last treatment. (f) The percentage of Th2 cell of the control group and the allergic asthma group before the first treatment and at the end of the last treatment. (g) The Th1/Th2 cell ratio of the control group and the allergic asthma group before the first treatment and at the end of the last treatment. Data are expressed as M ± SEMs (the control group: *n* = 8, the allergic asthma group: *n* = 10), all analyzed by the paired-samples *t*-test intragroup comparison; before the first treatment, all were analyzed by two independent samples *t*-test intergroup comparison; at end of the last treatment, Th1 cell and the Th1/Th2 cell ratio were analyzed by two independent samples *t*-test, and Th2 cell was analyzed by the nonparametric Mann–Whitney *U* test intergroup comparison. ^∗∗^*P* < 0.01, ^∗^*P* < 0.05.

(1) In the allergic asthma group, compared to baseline levels, the percentage of Th2 cell decreased (*P* = 0.027), and the Th1/Th2 cell ratio significantly increased (*P* = 0.027) after the last treatment. However, the percentage of Th1 cell did not change significantly by the end of the last treatment. (2) Compared with the control group, the percentage of Th2 cell was higher (*P* = 0.002), and the Th1/Th2 cell ratio was lower (*P* = 0.009) in the allergic asthma group; there was no significant difference in the percentage of Th1 cell before the first treatment. (3) Compared with the control group, the percentage of Th2 cell was higher (*P* = 0.026) in the allergic asthma group; there was no significant difference between Th1 cell and the Th1/Th2 cell ratio by the end of the last treatment, as shown in Figure 4.

### 4.5. RHAS Downregulated the Serum Levels of IL-4

**Figure 5:**
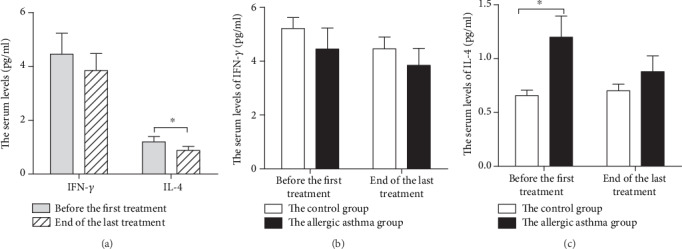
Effect of repeated herbal acupoint sticking on IFN-*γ* and IL-4 in the serum of allergic asthma participants. (a) The serum levels of IFN-*γ* and IL-4 before the first treatment and at the end of the last treatment in the allergic asthma group. (b) The serum levels IFN-*γ* of the control group and the allergic asthma group before the first treatment and at the end of the last treatment. (c) The serum levels of IL-4 of the control group and the allergic asthma group before the first treatment and at the end of the last treatment. Data are expressed as M ± SEMs (the control group: *n* = 13, the allergic asthma group: *n* = 26). The serum levels of IFN-*γ* were analyzed by the paired-samples *t*-test, and the serum levels of IL-4 were analyzed by the nonparametric Wilcoxon Signed-Rank Test *t*-test intragroup comparison; the sum levels of IFN-*γ* and IL-4 were analyzed by the nonparametric Mann–Whitney *U* test intergroup comparison. ^∗^*P* < 0.05.

(1) In the allergic asthma group, compared to baseline values, the serum levels of IL-4 decreased (*P* = 0.003), and the serum levels of IFN-*γ* did not change significantly by the end of the last treatment. (2) Compared with the control group, the serum levels of IL-4 were higher (*P* = 0.036) in the allergic asthma group, and there was no significant difference in the serum levels of IFN-*γ* before the first treatment. (3) Compared with the control group, there was no significant difference between the serum levels of IFN-*γ* and IL-4 by the end of the last treatment, as shown in Figure 5.

### 4.6. Safety and Adverse Events in RHAS

During the entire RHAS for allergic asthma, we observed no significant abnormalities in the routine blood workup or the liver and kidney function of the participants. Moreover, no serious adverse reactions were found.

## 5. Discussion

RHAS for treating allergic asthma, the most classic is the *Semen Brassicae* paste in the book *Zhang Shi Yi Tong*, was put forward by Zhang Lu, a famous doctor in Qing Dynasty. Since then, the therapy has been in use until now. The herbs, acupoints, and application methods in our study mainly referred to the records in this book, and relevant articles had been published in China [13]. RHAS can adjust the function of the organs and improve the condition; many clinical researches had proved that it can effectively prevent the recurrence of allergic asthma [14–16]. Our study also confirmed the function of its effects. Compared to before the first treatment, the recurrence number of allergic asthma participants decreased and the ACT scores increased in the allergic asthma group at end of the last treatment and 18 and 30 weeks of the trial, indicating that RHAS significantly reduced the number of recurrences, improved the allergic asthma participant's ability for daily activities and sleep status, and reduced the dyspnoea level and the use of emergency drugs, so as to ameliorate the control of allergic asthma participants and the quality of life. Gennaro et al. [17] found that the recurrence of allergic asthma is related to different seasons; therefore, we compared the 18 and 30 weeks of the trial with the same period of the previous year, excluded the factor of season, and found that the recurrence number of allergic asthma participants was less and the ACT scores were higher than the ones from the same time of the previous year. The curative effect of RHAS was verified again.

In a physiological condition, Th0 cells differentiate to Th1 and Th2 cells proportionally and keep their amount in a relative dynamic balance [6]. Allergic asthma is an inflammatory disease dominated by Th2 cells; patients have an unbalanced ratio of Th1/Th2 cell [2]; therefore, the recurrence of allergic asthma can be alleviated by inhibiting the Th2 cell response [18, 19]. By analyzing the changes in the Th1/Th2 cell ratio by flow cytometry, we found that RHAS decreased the percentage of Th2 cell and elevated the Th1/Th2 cell ratio. Compared with the control group, our findings showed that while the percentage of Th2 cell in the allergic asthma group was higher at end of the last treatment, it did alter the Th1/Th2 cell ratio, bringing it closer to ratio seen in healthy participants, thereby effectively alleviating recurrence. In addition, in this study, we found that the percentage of Th1 in the allergic asthma group was like the control group before the first treatment; RHAS treatment also did not change this value significantly.

To investigate the role of RHAS in alleviating allergic asthma recurrence, we examined the levels of IFN-*γ* and IL-4 cytokines associated with airway inflammation in serum. IL-4 plays a key role in the differentiation of Th0 cell into Th2 cell and is also important for stimulating B cell production and the release of IgE antibody [20–22]. Th1 cell has the opposite effect, and IFN-*γ* is involved in antagonizing Th2 cell response and IgE synthesis to inhibit allergic asthma progression [23, 24]. Therefore, in order to study the immunoregulatory mechanism of RHAS in the treatment of allergic asthma, we measured the serum levels of IgE, IFN-*γ*, and IL-4 by ELISA. Our results show that RHAS significantly inhibited the release of IgE, while, compared to the control group, the serum levels of IgE were higher in the allergic asthma group. Moreover, RHAS significantly reduced the serum levels of IL-4, which was about the same as healthy participants after treatment. As for the serum levels of IFN-*γ*, it was at the same level as that of healthy people before treatment; this result also confirmed the percentage of Th1.

In terms of the recurrence number of the allergic asthma participants and the ACT score, allergic asthma also improved at 18 and 30 weeks of the trial, which may be related to the changes of the levels of IgE, Th1 and Th2 cells, IFN-*γ*, and IL-4 cytokine. However, the above laboratory tests were not carried out at 18 and 30 weeks of the trial, because these indicators were associated with the state of the participants. From a clinical point of view, there are many intervention factors that are out of control, such as whether to take drugs or receive other treatment and the occurrence of other diseases. In the future, we will try our best to improve the experimental design and eliminate the influence of other interference factors in order to obtain objective and real results.

In summary, this study demonstrated that RHAS could improve systemic immune response by elevating the Th1/Th2 cell ratio and decreasing the levels of IgE and IL-4, thereby effectively preventing the recurrence of allergic asthma. This study proved the efficacy of a convenient, effective, and hormone-independent method for allergic asthma treatment and enhanced our understanding of the specific mechanism of traditional Chinese medicine treatment of allergic asthma.

## Figures and Tables

**Table 1:** Baseline levels of the study participants.

		The allergic asthma group	The control group	*x* ^2/*t*/*Z*^	*P* value
Clinical parameters	*N*	38	13		
Age, mean (SD)		52.24 (12.56)	50.92 (13.76)	*t* = 0.318	0.752
Sex, male/female		14/24	4/9	*x* ^2^ = 0.156	0.692
The course of disease, mean (SD)		14.13 (11.71)			
^1^Recurrence number of allergic asthma participants, yes/no		18/20			
^2^ACT score (SD)		20.50 (3.49)			

^1^Recurrence number of allergic asthma participants mainly depended on whether participants had a relapse; ^2^ACT score: asthma control test, higher score denoted better allergic asthma-related quality of life. SD: standard deviation; *N*: number.

**Table 2:** Schedule of study procedures.

Time point	Informed consent	Vital signs^1^	Safety^2^	Adverse event^3^
W1				
W0	+	+	+	+
W1		+		+
W2		+		+
W3		+		+
W4		+		+
W5		+		+
W6		+	+	+
W18				+
W30				+

^1^Vitals signs: heights, weight, temperature, blood pressure, and heart rate. ^2^Safety: blood routine and kidney and liver function. ^3^Adverse event: occurred to participants regardless of relationship to the trial intervention.

**Table 3:** Recurrence number of allergic asthma participants.

	End of the last treatment (*n* = 38)	18 weeks of the trial (*n* = 38)	30 weeks of the trial (*n* = 38)	The same period last year of 18 weeks of the trial (*n* = 38)	The same period last year of 30 weeks of the trial (*n* = 38)
Yes, *n*	8	9	9	18	24
No, *n*	30	29	29	20	14
*x* ^2^	5.846	4.653	4.653	4.653	12.051
*P* value	^1^0.016	^1^0.031	^1^0.031	^2^0.031	^3^0.001

Recurrence number of allergic asthma participants mainly depended on whether the participants had a relapse. ^1^Compared with the first treatment. ^2^Compared with the same period last year period last year of 18 weeks of the trial. ^3^Compared with the same periods last year of 30 weeks of the trial.

## Data Availability

All relevant data are contained within the paper and its Supporting Information file.

## Abbreviations

ACT: Asthma control test
IgE: Immunoglobulin E
Th1: T helper 1 cell
Th2: T helper 2 cell
IFN-*γ*: Interferon-*γ*
IL-4: Interleukin-4
W: Week
*N*: Number
M: Mean
SD: Standard deviation
SEM: Standard error of mean
GINA: Global initiative for asthma.

## Supplementary Materials

Supplementary Materials Dataset S1: original dataset. (XLSX).
